# Negative affective bias in depression following treatment with psilocybin or escitalopram – a secondary analysis from a randomized trial

**DOI:** 10.1038/s41398-025-03693-w

**Published:** 2025-11-13

**Authors:** Marieke A. G. Martens, Bruna Giribaldi Cunha, David Erritzoe, David Nutt, Robin Carhart-Harris, Catherine J. Harmer

**Affiliations:** 1 Department of Psychiatry, University of Oxford, Oxford, UK; 2 Oxford Health NHS Foundation Trust, Oxford, UK; 3 Centre for Psychedelic Research, Division of Psychiatry, Department of Brain Sciences, Faculty of Medicine, Imperial College London, London, UK; 4 Department of Neurology, Psychiatry and Behavioural Sciences, University of California San Francisco, San Francisco, CA USA

**Keywords:** Depression, Human behaviour

## Abstract

Recent clinical trial data suggests that ratings on depression scales are lowered after psilocybin therapy compared to placebo, though it is unclear what neuropsychological mechanisms underpin these effects. This study compared psilocybin, with an established antidepressant, escitalopram, to investigate whether there are shared or distinct effects on emotional information processing. Patients with long-standing moderate-to-severe depression were randomly and double-blindly assigned in a 1:1 ratio to receive either 1) two doses of 25 mg of psilocybin, 3-weeks apart, plus 6-weeks of daily placebo (psilocybin group N = 30); or 2) two doses of 1 mg of psilocybin 3-weeks apart plus 6-weeks of daily oral escitalopram (escitalopram group N = 29); all patients received the same psychological support. Behavioural measures of affective bias as well as subjective measures of depression were collected at baseline and at the primary 6-week endpoint, using an established computerised task (Facial Emotion Recognition Task) and Quick Inventory of Depressive Symptomatology, respectively. Change in affective bias was further correlated with change in depression scores measured concurrently as well as at 1-month post-trial follow-up (week-10), corrected for baseline depression severity. Negative bias in facial expression recognition decreased after both treatments to a comparable level. Concurrently, change in negative affective bias was not associated with change in depression. Longitudinally, a decrease in the misclassification of positive faces as negative was associated with a decrease in depression scores at week-10 for the escitalopram group only. Therefore, a more positive behavioural bias in emotional processing was seen following psilocybin and citalopram compared to baseline. This highlights the potential for at least some overlap in cognitive mechanisms across two distinct treatments, which is noteworthy given the short dosing regimen with psilocybin.

## Introduction

Even though effective treatments for depression are available, current first line antidepressant strategies (like the selective serotonin reuptake inhibitors, SSRIs) remain burdened by partial efficacy, less-than-perfect side-effects profile, and a slow onset of therapeutic action [1, 2]. Within this context, there is a pressing need to develop new treatments for depression that target novel brain mechanisms and ideally have a faster onset of therapeutic action. For this reason there has been growing interest in psychedelics including psilocybin - the pro-drug of psilocin, the active ingredient in so-called ‘magic mushrooms’ [3].

As with other traditional psychedelic substances, the main effects of psilocybin occurs through serotonin 5-hydroxytryptamine type 2 A (5-HT2A) receptor agonism, a key pathway implicated in depression which has been proposed to be (somewhat) distinct from the effects of SSRIs [4–8]. Previous trials have shown that psilocybin may have antidepressant properties. For example, in a randomized clinical trial of 24 participants with major depressive disorder (MDD), participants who received immediate psilocybin-assisted therapy compared with delayed treatment showed improvement in blinded clinician rater–assessed depression severity and in self-reported secondary outcomes through the 1-month follow-up [9]. Furthermore, as part of the trial from which this current paper is derived, depression scores fell with both psilocybin and escitalopram treatment compared to placebo over a 6-week period in a trial involving 59 patients with long-standing, moderate-to-severe MDD [10, 11]. In a recent Phase 2 trial, participants with difficult to treat depression taking a single 25 mg dose of psilocybin showed lower depression ratings compared to those who took a 1 mg dose over 3 weeks [12] and two further trials have also supported the promise of psilocybin therapy for depression [13, 14]. In addition, several meta-analyses have also been conducted which provide preliminary evidence for a reduction in symptoms of depression after psilocybin-assisted psychotherapy [15–18]. However, it is unclear what neuropsychological mechanisms underpin these effects, and whether psilocybin has effects on emotional information processing that are comparable with those of conventional antidepressant drugs, like SSRIs [19]. Exploring the neurocognitive effects of novel potential treatments can help unpick mechanisms and are less susceptible to placebo or expectation effects [20]; a real issue in psychoactive drug trials, where blinding is easily corrupted due to participants correctly guessing treatment allocation due to detectable drug effects [21–23].

Emotional processing refers to the way in which humans attend, interpret and remember emotional information. Biases in how emotional information is processed have been reported across many psychiatric disorders including MDD. For example, patients with MDD have a tendency to interpret social cues as more negative (including recognising fewer happy facial expressions compared to non-depressed controls) [24–27]. According to Beck’s cognitive model of depression [28, 29] these biases are believed to play a fundamental role in the development and maintenance of MDD. An experimental medicine framework we have developed based on a cognitive neuropsychological model for depression employs behavioural and neural investigations of the circuitry involved in the experience of depression and the mode of action of antidepressants [19, 30]. This model suggests that successful treatments for MDD, regardless of their specific pharmacology, exert their clinical effects via a shared ability to (sub-) acutely change the balance of negative versus positive emotional processing. In particular, this is typically seen as a shift toward more positive vs negative processing, for example an improvement in the accuracy for recognising happy emotional expressions following antidepressant compared to placebo administration, contrary to the effects of depression itself [27, 31]. Notably, these effects on emotional information processing can be measured long before improvements in the clinical state normalise and are associated with later therapeutic gain [32–35]. As such, emotional bias change is believed to take time to lead to changes in mood, as interaction with the environment is needed to feel the effects of these bias changes. This model together with the task used in this study (see below) has further developed into a human emotion model to screen and understand novel treatments for depression in development, e.g., such as used in the study by Post et al., [36].

The current study therefore sought to investigate whether psilocybin had similar effects on emotional processing as conventional antidepressant drugs. We compared the effects of psilocybin with the antidepressant escitalopram on emotional cognition using a task sensitive to antidepressant drugs the Facial Expression Recognition Task (FERT), part of the Emotional Test Battery (ETB) [27]. The FERT has been widely used, is well-validated, and has been shown to be sensitive to and specific for pharmacological manipulations using conventional antidepressants [24, 37]. This was performed as part of the Carhart-Harris et al., 2021 [10] study described below in Methods. We predicted that both psilocybin and escitalopram would induce a more positive behavioural bias in emotional information processing following treatment and these effects would be associated with subsequent therapeutic response. We expected that emotional bias would be associated with subsequent, rather than concurrent, depression scores because of the need for interactions between emotional bias and environmental stimuli to occur.

## Methods

### Participants, study procedures and measures

In a phase 2, double-blind, randomized, controlled trial involving patients with long-standing, moderate-to-severe major depressive disorder (previously described in full in [10] ClinicalTrials.gov number, NCT03429075) psilocybin was compared with escitalopram, a selective serotonin-reuptake inhibitor, over a 6-week period. The trial took place from January 2019 through March 2020 at the National Institute for Health Research [NIHR] Imperial Clinical Research Facility [CRF]. Eligibility criteria, sample size calculation, as well as randomization procedure were described in full in [10].

In short, eligible patients were assigned in a 1:1 ratio to receive two separate doses of 25 mg of psilocybin 3 weeks apart plus 6 weeks of daily placebo (psilocybin group) or two separate doses of 1 mg of psilocybin 3 weeks apart plus 6 weeks of daily oral escitalopram (escitalopram group; 10 mg escitalopram first 3 weeks, 20 mg the following 3 weeks); all patients received the same psychological support before, during and after the two psilocybin dosing sessions.

Members who were not part of the research team performed the randomization. At visit 1 (baseline) all the patients attended a preparatory therapeutic session, completed a range of questionnaires (including the Quick Inventory of Depressive Symptomatology (QIDS-SR-16)), a battery of cognitive and affective processing tasks (including FERT, presented here, primary outcome measure) and underwent functional MRI [8, 38]. At visit 2, which occurred 1 day after visit 1, the patients received their treatment. To minimise expectation effects all patients were informed that they would receive psilocybin without disclosing the dose. Visit 3 occurred 1 day after dosing day 1 and included a psychological debriefing or ‘integration’, consisting mainly of open, attentive and compassionate listening. An additional debriefing by telephone or video call occurred 1 week later. At visit 4, which occurred 3 weeks after dosing-day 1, the patients received their second dose of psilocybin or placebo (dosing-day 2), and at visit 5 (the next day), another psychological integration session was held. Three weeks after visit 5, the patients returned for their final trial visit (visit 6 = primary post-treatment endpoint) for the assessment of the primary outcome. The structure of this visit was similar to that of visit 1. After week 6, the patients were followed monthly for 6 months by the investigators, including monthly administration of the QIDS-SR-16 and a remote interview at 1 month (10 weeks).

### Ethics approval and consent to participate

All methods were performed in accordance with the relevant guidelines and regulations, including the principles of Good Clinical Practice. A Schedule 1 drug license from the U.K. Home Office was obtained by the investigators, and the trial was sponsored by Imperial College London. Ethical approved was granted by the Brent Research Ethics Committee, the U.K. Medicines and Healthcare Products Regulatory Agency, the Health Research Authority, the Imperial College London Joint Research Compliance and General Data Protection Regulation Offices, and the risk assessment and trial management review board at the trial site (the National Institute for Health Research [NIHR] Imperial Clinical Research Facility [CRF]). All patients provided written informed consent.

### Behavioural tasks

The facial emotion recognition task (FERT) is part of the Emotional Test Battery designed to assess the processing of a variety of emotionally valenced stimuli. It is sensitive to negative biases in emotional processing observed in depression and to the early effects of antidepressants on emotional processing [27]. This task was performed once at baseline and once after 6 weeks post the first (and hence 3 weeks post the second) psilocybin treatments, and therefore after 6 weeks of escitalopram in that group.

### Facial expression recognition task (FERT)

The FERT was previously described in full (for example see [39]. In short, participants were presented with pictures of facial expressions displaying one of the six basic emotions (i.e., anger, disgust, fear, happiness, sadness and surprise) or neutral. Each emotion was displayed at 10 morphed intensity levels from “neutral” (i.e. 0% emotion) to “full intensity” (i.e. 100% emotion), based on a previously described procedure by [40]. In total 250 stimulus presentations (6 emotions × 10 intensities × 4 examples + 10 neutral faces) were presented in random order for approximately 500 ms each, followed by a blank black screen. Participants were instructed to identify each emotion as quickly and as accurately as possible via a keyboard press. The main outcomes of interest were accuracy, misclassifications (i.e., number of faces misclassified as a particular emotion), and averaged reaction time for correct classifications. To complement the analysis an alternative approach was also used comparing groups on signal detection theory measures (i.e., target sensitivity and response bias (higher scores mean less response bias), calculated following Grier, 1971 [41].

### Effects on emotional cognition

Emotions were grouped into negative (anger, disgust, fear, sadness) and positive (happy and surprise). An improvement of negative bias was defined as a statistically significant reduction over time in accuracy, misclassifications), and speed for the classification of negative vs positive emotions.

### Subjective measures

As described in full in [10], the primary clinical outcome measure of the original trial was the change from baseline in the score on the 16-item Quick Inventory of Depressive Symptomatology–Self Report (QIDS-SR-16) at 6 weeks. These scores were used in the correlation analysis (see below). For effects of psilocybin and escitalopram on the QIDS-SR-16 see [10].

### Analysis of behavioural data

Analysis of behavioural data was conducted in IBM SPSS (for Mac, version 29.0). FERT data was analysed using a three-way mixed ANOVA with group (escitalopram or psilocybin) as the between-subjects factor and visit (baseline or post-treatment) and emotion/valence as within-subjects factors, respectively. Effects of interest were group × time interactions and group × emotion × time interactions. Planned-post hoc comparisons were explored regardless of overall significance. Where the assumption of sphericity was broken, Greenhouse-Geisser correction was used.

Partial correlations were conducted on change-from-baseline scores between emotional cognitive measures and depression scores measured with the QIDS-SR-16 at 6 weeks (concurrent analysis) as well as at one-month follow-up (10 weeks, longitudinal analysis) controlling for baseline For the longitudinal correlation analysis patients were excluded if they took psilocybin during the follow-up period when they were in the escitalopram group (n = 4) or SSRIs when in the psilocybin group (n = 8).

Additional sensitivity analyses were conducted which only included the participants who followed the full protocol (per protocol participants n = 46; n = 21 escitalopram, n = 25 psilocybin).

## Results

**Fig. 1:**
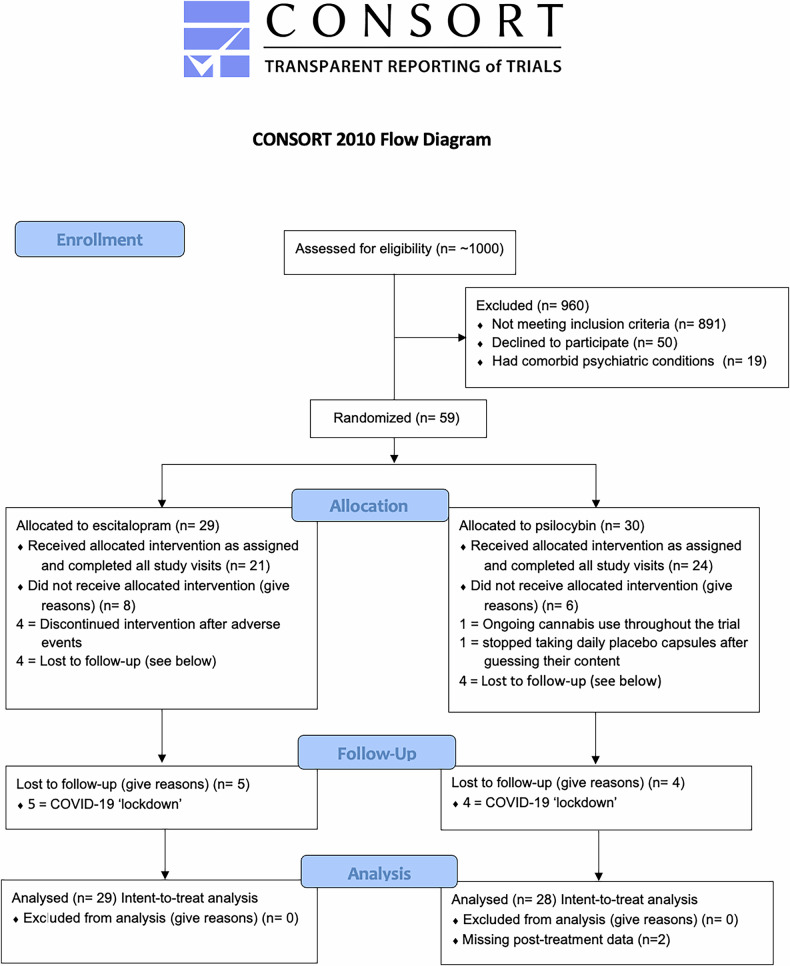
CONSORT diagram to show flow of participants through the study.

Sociodemographic, clinical, and personality characteristics of participants of the final sample (n = 59; n = 29 escitalopram, n = 30 psilocybin) were presented in Table 1 of the original paper [10]. In the escitalopram group, 8 of 29 patients did not complete the protocol requirements: 4 stopped taking their escitalopram capsules because of adverse events, and 4 missed psilocybin dosing-day 2 and subsequent visits owing to restrictions related to coronavirus disease, 2019 (Covid-19). Two patients in the escitalopram group stayed on the 10 mg dose: one guessed that the capsules contained escitalopram and reduced the dose by half (from 20 mg to 10 mg) because of perceived adverse events; and the other misunderstood instructions to take 2 tablets after dosing day 2 so only took 1. A reduction in the escitalopram dose to 10 mg was permitted in the protocol because it reflects clinical practice. In the psilocybin group, 4 of 30 patients missed dosing-day 2 and subsequent visits because of Covid-19–related restrictions. For one patient dosing day 2 was on week 5 rather than week 3 due to illness. After the end of the trial, it was revealed that one patient in the psilocybin group had been using cannabis regularly throughout the trial, and another patient stopped taking placebo tablets around mid-week 5 without telling the team. All the patients who had undergone randomization are included in an intention-to-treat analysis. See also Fig. 1.

### Facial Emotion Recognition Task (FERT)

#### Differences between psilocybin and escitalopram

**Fig. 2:**
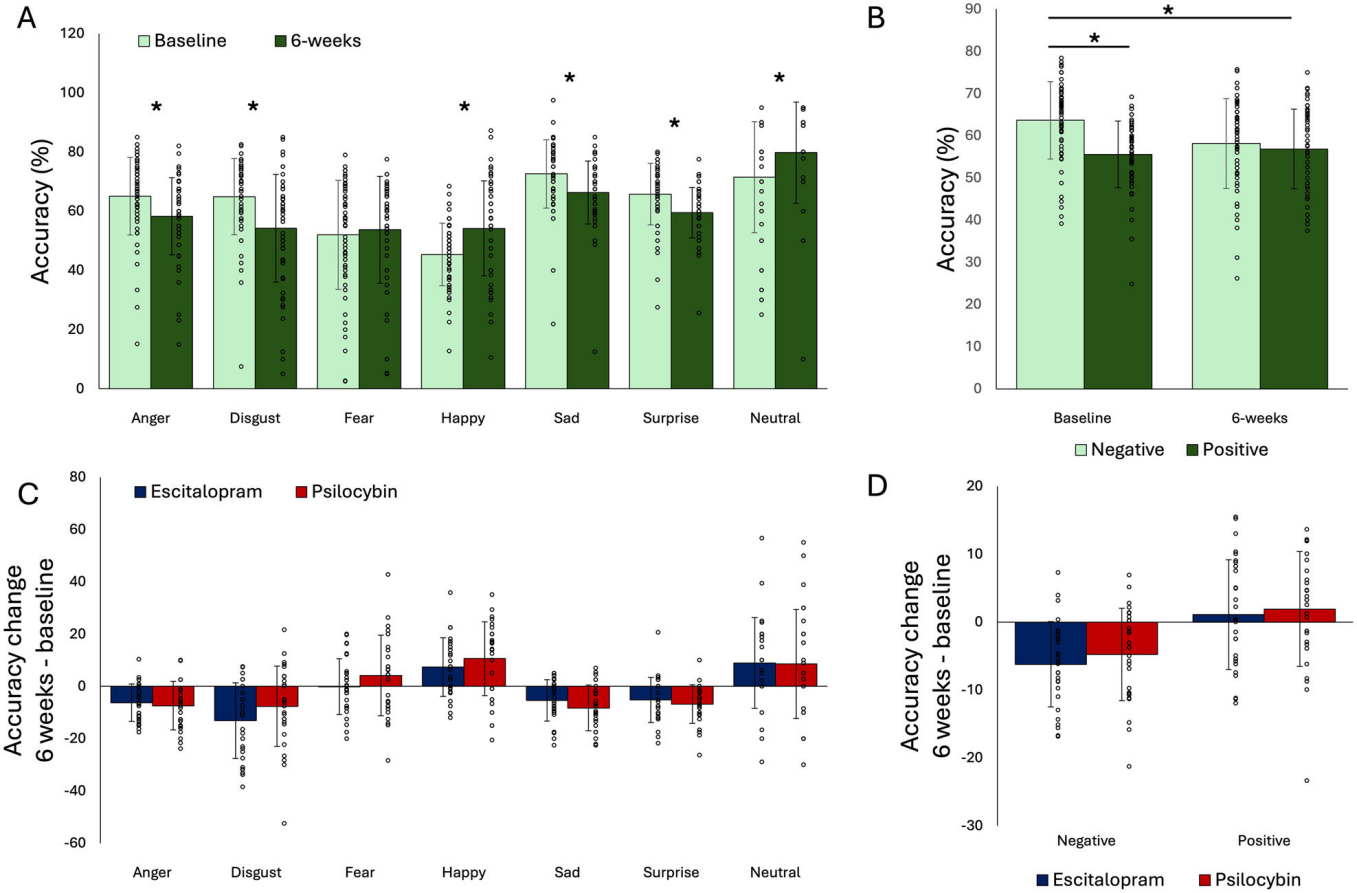
Accuracy performance on facial recognition (FERT). (**A**) Bars show difference in accuracy (%-correct) across groups over time for all seven emotions and (**B**) for emotions grouped into negative and positive emotions. (**C**) Change from baseline accuracy scores (6 weeks minus baseline) presented for escitalopram and psilocybin groups separately for all seven emotions and (**D**) for emotions grouped into negative and positive emotions. * p < 0.05 post-hoc difference. Error bars present standard deviation (SD).

**Fig. 3:**
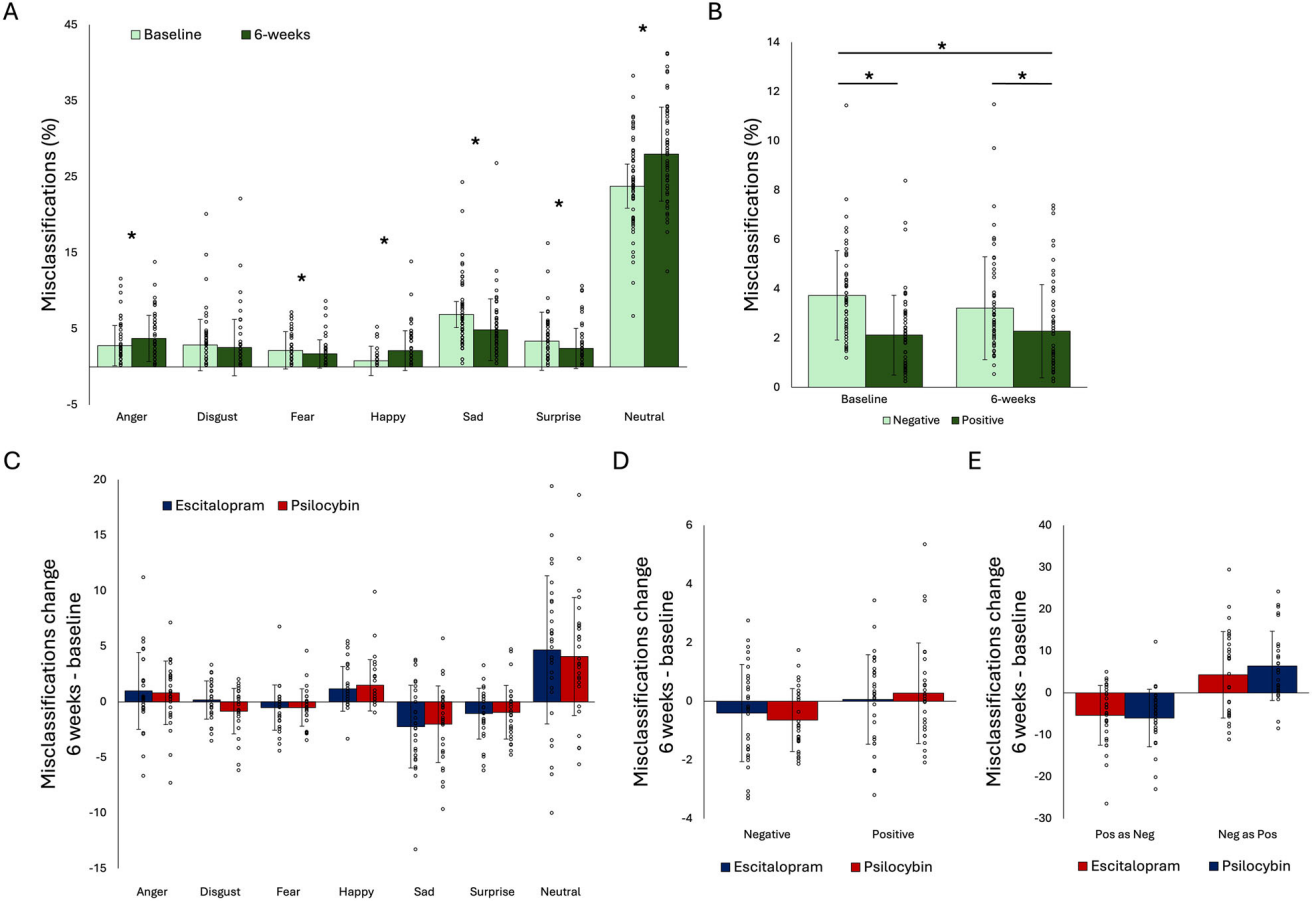
Misclassifications on the FERT. (**A**) Percentage misclassifications across groups over time for all seven emotions and (**B**) emotions grouped into negative and positive. (**C**) Change from baseline in % misclassifications for psilocybin and escitalopram groups for all seven emotions and (**D**) emotions grouped into negative and positive. (**E**) Change from baseline in the mean number of misclassifications of positive faces as negative, and negative faces as positive. * p < 0.05 post-hoc difference. Error bars present standard deviation (SD).

There were no statistically significant differential effects of treatment (i.e., group × time interaction or group × time × emotion interaction) on any of the task outcomes: accuracy F’s < 0.52, p’s > 0.475; misclassifications F’s < 0.11, p’s > 0.746; reaction times F’s < 2.23, p’s > 0.141; response bias F’s < 0.89, p’s > 0.349; target sensitivity F’s < 0.58, p’s > 0.448) (See also Supplementary Table S1 and Figs. 2 and 3).

When analysing the number of misclassifications of positive faces as either negative or neutral, or negative faces as either positive or neutral, there were no statistically significant time × group interactions either (F’s < 0.72, p’s > 0.399) (See Supplementary Table S1 and Fig. 2).

#### Effects of treatment over time

Across groups, there was a statistically significant negative bias at baseline for accuracy, which improved over time, reflected in a statistically significant time by emotion interaction (F(1,55) = 30.44, p < 0.001, η2 = 0.36), Fig. 2). Post-hoc comparisons showed there was a statistically significant effect of testing session for negative emotions (t(56) = 6.39, p < 0.001, d = 0.85, 95% CI: 3.79-7.25) but not positive ones (t(56) = -1.381, p = 0.174, 95% CI: -0.69 – 3.70) with patients being less accurate for negative emotions post treatment compared with baseline.

A similar pattern of results was found for misclassifications, where negative bias was reduced after 6 weeks compared to baseline (Fig. 3). There was a statistically significant time by emotion interaction (F(1,55) = 5.17, p = 0.027, η2 = 0.086) where there was a statistically significant effect of testing session for negative (t(56) = 2.81, p < 0.001, d = 0.37, 95% CI: 0.15 – 0.89) but not positive (t(56) = -0.78, p = 0.439, 95% CI: -0.59 – 0.26) emotions. Participants showed less misclassifications for negative emotions after treatment compared with baseline. Looking at the direction of these misclassifications, there was both a decrease in positive for negative substitutions over time (F(1,55) = 37.61, p < 0.001, η2 = 0.41) as well as an increase for negative to positive substitutions (F(1,55) = 18.67, p < 0.001, η2 = 0.25) (Fig. 3E).

Across groups there was a statistically significant time by emotion interaction on reaction times for accurate trials (F(1,55) = 5.11, p = 0.028, η2 = 0.085). Post-hoc comparisons showed patients became quicker post-treatment compared with baseline for negative emotions (t(56) = 3.28, p = 0.002, d = 0.43, 95% CI: 29.9 – 124.0), and even quicker for positive emotions (t(56) = 5.57, p < 0.001, d = 0.74, 95% CI: 90.7 – 192.7).

Statistically significant time by emotion interactions were found across groups for response bias (F(1,55) = 7.50, p = 0.008, η2 = 0.12) and target sensitivity (F(1,55) = 29.45, p < 0.001, η2 = 0.35) as well. Post-hoc comparisons showed that response bias (higher score means less response bias) was reduced for negative emotions post-treatment compared with baseline (t(56) = -4.04, p < 0.001, d = -0.54, 95% CI: -0.07 – -0.03) but not for positive emotions (t(56) = 0.56, p = 0.586, 95% CI: -0.02 – 0.04). Target sensitivity was lower for negative emotions post-treatment compared with baseline (t(56) = 4.85, p < 0.001, d = 0.64, 95% CI: 0.009 – 0.02) but not positive emotions (t(56) = -1.09, p = 0.281, 95% CI: -0.009 – 0.003). See supplementary Table S1.

#### Correlations

Correlations between negative bias measures and subsequent clinical response at both 6 weeks (concurrent analysis) and one month later follow-up (longitudinal analysis) were computed, controlling for baseline depression severity.

**Fig. 4:**
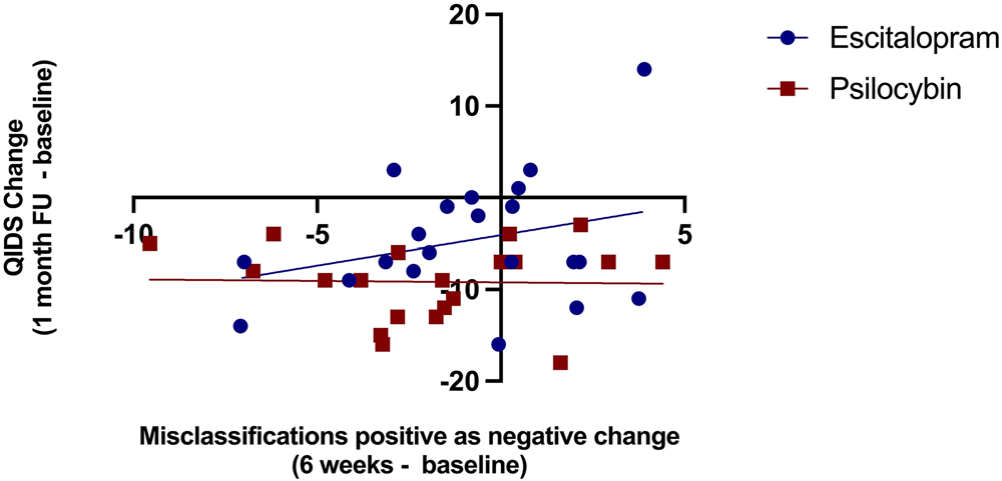
Correlations change-from-baseline scores between emotional cognitive measures and depression scores at one-month follow-up, controlling for baseline.

Concurrently, there were no associations between change in negative affective bias (week 6 minus baseline) and subsequent therapeutic gain (week 6 minus baseline) as measured by the QIDS (Supplementary Table S2). Longitudinally, there was a statistically significant positive correlation between the change in misclassifications of positive faces as negative at week 6 (negative bias) and depression scores one month later (week 10 minus baseline) (r(18) = 0.498, p = 0.025) in the participants receiving escitalopram, whereby the week 6 decrease in negative bias was associated with later decrease in depression scores. This suggests that change in the processing of positive faces is associated with subsequent therapeutic gain following treatment with escitalopram, as predicted by the environment x bias model. By contrast this association was not statistically significant for participants receiving psilocybin (r(17) = -0.195, p = 0.423) and these correlation coefficients differed statistically significantly from each other (Z = 2.20, p = 0.028). See Fig. 4. No other statistically significant associations were seen, see Supplementary Table S3.

#### Sensitivity analysis

Additional sensitivity analyses were conducted that only included the per protocol participants (n = 46; n = 21 escitalopram, n = 25 psilocybin). Results remained similar, though the longitudinal correlation effect was smaller. See Supplementary Table S4, S5.

## Discussion

This study compared the effects of psilocybin with the conventional antidepressant escitalopram over a 6-week trial period on emotional cognition using a task that has previously been shown to be sensitive to effective antidepressant treatments for depression. In line with our predictions, both psilocybin and escitalopram treatment were associated with a more positive (or less negative) behavioural bias in emotional information processing. This was detected as a statistically significant decrease in accuracy for recognising negative faces from baseline to week 6, as well as a statistically significant decrease in misclassifying other faces as negative compared to baseline. There were no statistically significant differences between the two treatments on these measures.

To our knowledge, this is the first study to compare the effect of psilocybin with a traditional SSRI on behavioural measures of emotional processing. Our study adds further evidence to psilocybin’s antidepressant potential. Previously, both psilocybin and escitalopram were found to be equally successful in reducing depressive symptoms according to the primary clinical outcome measure [10], though see a Bayesian re-analysis supporting more nuanced inferences [42] as well as a systematic critique of the primary outcome [11]. Despite these findings, it is unclear what neuropsychological mechanisms underpin psilocybin’s effects, though some recent evidence-informed models have been proposed centred on an increase in neural [43] or brain network flexibility [8].

Conventional antidepressants like SSRIs are thought to within a few days change the balance of positive versus negative emotional processing in the brain but it is only with time and in interaction with social and emotional experiences that effects on mood and subjective experience can be seen [24]. Studies comparing psilocybin with placebo in in difficult to treat depression also showed some evidence for psilocybin to improve behavioural measures of affective recognition, albeit valence independent [44]. In a sample of non-depressed healthy volunteers, psilocybin enhanced subjective current positive mood scores, and decreased the recognition and encoding of negative facial expressions. These effects were abolished with coadministration of a 5-HT2A antagonist (ketanserin) [45, 46]. There is some further evidence emerging from human imaging studies, however these are heterogeneous with differences in population studied, analysis-methods and dosing regimens used [47].

Our behavioural findings are interesting in this context as psilocybin and escitalopram had divergent effects at the neural level [38]. Specifically, escitalopram was associated with reduced Blood Oxygenation Level Dependent (BOLD) responses across a network of areas, including the amygdala, to facial expressions, whereas there was no overall effect of psilocybin. Other studies have shown psilocybin to acutely and sub-acutely alter amygdala activity and connectivity during the viewing of emotional stimuli [48–54], with some evidence that increased amygdala responsivity to emotional faces (one day post treatment) is predictive of clinical improvement in depressed patients in an open label study [52]. Also amygdala reactivity in response to negative stimuli correlated with the psilocybin-induced increase in positive mood state in healthy volunteers [49].

Moreover, during rest, psilocybin has also been found to alter within and between network functional connectivity as well as the coupling profile of a number of regions [43, 55–59]. An additional consideration across studies is the distinction between acute and post-acute brain effects: these appear to differ on certain metrics [43, 47, 57] though not necessarily all. For example, consistent decreases in network modularity have been seen acutely with LSD [60] but post acutely with psilocybin therapy for depression [8].

Different neuropsychological theories on the antidepressant effects of psilocybin have emerged from these neuroimaging findings, including psilocybin reviving emotional responsiveness believed to allow patients to reconnect with their emotions [52, 54, 61] as well as psilocybin increasing cognitive flexibility [43, 47, 53, 62, 63] both resulting in more immediate effects on mood. There could be several explanations for why the therapeutic effect of SSRI treatment requires a number of weeks to become clinically apparent whilst the effects of psilocybin occur within days (in addition to possible placebo and expectation effects). For example, psilocybin is a direct serotonin agonist whilst SSRIs are indirect, which could mean that the increase of synaptic serotonin takes time to translate into increased post-synaptic signalling [5]. Another potential explanation is that the more positive emotional bias after SSRI treatment requires positive interaction with the psychosocial milieu to improve mood, which takes time, whilst psilocybin has been proposed to affect responses to previously established negative memories and therefore requires less time than when new information processing needs to take place [64]. Alternatively, a certain level of cognitive flexibility is required for expectations about negative and positive events to be corrected, which may take longer for conventional treatments [63]. This hypothesis is supported by evidence that psilocybin has faster effects on novel protein synthesis and neural plasticity than is typically seen with SSRIs [65].

Despite the suggested mechanistic differences between the two treatments described above, this current study did not find any emotional cognitive behavioural differences between psilocybin and escitalopram. Both treatments were associated with a more positive (or less negative) emotional processing bias. As the current study design did not measure the immediate (i.e., acute / early within hours) effects of both escitalopram nor psilocybin on the FERT, it is not yet possible to say whether these drugs share some direct mechanisms of action. There was no correlation between negative bias change and depression change measured concurrently. However, longitudinally negative bias change was found to correlate with subsequent persistent clinical response following escitalopram, but not psilocybin. It is important to note that while changes in negative bias have been found to predict later clinical mood this has typically been measured much earlier than in the current study, before changes in symptoms. However, the stronger correlation at week 10 (compared to week 6) would be consistent with greater opportunities to learn from environment x bias interactions across time. It is interesting to note that correlations for psilocybin, albeit not statistically significant, tended to be in the opposite direction (reduction in symptoms associated with a more negative emotional processing bias, across metrics). This suggests that while, consistent with previous reports, change in negative bias may be an important mechanism of action for escitalopram, it may be less important (or just part of a range of changes) which mediate response to psilocybin. In particular, this could also include functional unblinding effects which were not assessed in the trial. However, there was no evidence that expectancy bias in the psilocybin arm predicted therapeutic response [66]. Further studies exploring the early effects of both compounds in relation to later changes would be needed to resolve this question.

The results reported here should be interpreted in the light of several strengths and limitations pertaining to the study. The assay that was employed has been widely used, is well-validated, and has been shown to be sensitive to and specific for pharmacological manipulations using conventional antidepressants [24]. Experimental medicine models are furthermore less susceptible to placebo effects [20] which add weight to the therapeutic effects of psilocybin. However, the study design currently applied lacked a simple inert placebo control group. In both trial groups, the scores on the depression scales at week 6 were numerically lower than the baseline scores, but the absence of a placebo group in the trial limits conclusions. Furthermore, an inadvertent sample bias (in favour of psilocybin) may have biased the trial sample towards patients who could receive psilocybin without unacceptable side effects and were especially open to receiving this drug (e.g. many expressed a preference for psilocybin over escitalopram). Also practice effects might have obscured valence-specific effects in task performance, as participants might have learned how to respond to stimuli efficiently during the first visit, though such learning effects are not usually described with this procedure. Last, the sample may have been underpowered to detect between group differences.

Future studies should incorporate an active placebo group to overcome potential blinding effects. Moreover, further studies exploring the early effects of both psilocybin and escitalopram on emotional processing bias in relation to later changes would be important to disentangle key mechanisms of antidepressant drug action.

In sum, both psilocybin and escitalopram treatment were associated with a more positive, or less negative, behavioural bias in emotional information processing compared to baseline scores. Further research is warranted to explore whether this could constitute a relevant mechanism by which psilocybin reduces symptoms of depression and whether these emotional bias changes precede changes in symptoms.

## Supplementary information

Supplementary Material

## Data Availability

Anonymised subjective and behavioural data that support the findings of this study are available from the corresponding author MM or RCH upon reasonable request.
