# Supplementary material for: Negative affective bias in depression following treatment with psilocybin or escitalopram – a secondary analysis from a randomized trial
Source: Transl Psychiatry. 2025 Nov 13;15:502. doi: 10.1038/s41398-025-03693-w (PMC12644704; doi:10.1038/s41398-025-03693-w)
Supplement: Supplementary file 1 — Supplementary Material [file 41398_2025_3693_MOESM1_ESM.docx]

**Negative affective bias in depression following treatment with psilocybin or escitalopram – a secondary analysis from a randomized trial**

Results

Table S1. Behavioural measures of emotional processing at baseline and after 6 weeks (FERT). Values represent means with standard error of the mean in parentheses.

|  | **Escitalopram** | | | | **Psilocybin** | | | |
| --- | --- | --- | --- | --- | --- | --- | --- | --- |
| **FERT** | **Baseline** | | **6-weeks** | | **Baseline** | | **6-weeks** | |
| **Accuracy (%)** | **Mean** | **SEM** | **Mean** | **SEM** | **Mean** | **SEM** | **Mean** | **SEM** |
| **Anger** | 65.13 | 2.60 | 58.92 | 2.71 | 64.94 | 2.43 | 57.55 | 2.15 |
| **Disgust** | 64.01 | 2.85 | 50.88 | 3.32 | 65.36 | 1.94 | 57.72 | 3.42 |
| **Fear** | 53.29 | 3.59 | 53.15 | 3.48 | 50.23 | 3.40 | 54.38 | 3.33 |
| **Happiness** | 46.31 | 2.37 | 53.69 | 3.25 | 44.08 | 1.55 | 54.70 | 2.77 |
| **Sadness** | 70.39 | 2.31 | 64.98 | 2.40 | 76.00 | 1.55 | 67.72 | 1.46 |
| **Surprise** | 64.84 | 1.77 | 59.65 | 1.35 | 66.15 | 2.16 | 59.36 | 1.84 |
| **Neutral** | 74.09 | 3.15 | 83.01 | 2.57 | 67.90 | 3.89 | 76.48 | 3.71 |
| **Negative** | 63.21 | 1.99 | 56.98 | 2.17 | 64.13 | 1.40 | 59.34 | 1.80 |
| **Positive** | 55.58 | 1.71 | 56.67 | 1.90 | 55.11 | 1.22 | 57.03 | 1.65 |
|  |  |  |  |  |  |  |  |  |
| **Misclassifications (%)** |  |  |  |  |  |  |  |  |
| **Anger** | 3.29 | 0.56 | 4.27 | 0.62 | 2.38 | 0.43 | 3.19 | 0.50 |
| **Disgust** | 2.83 | 0.77 | 3.00 | 0.87 | 2.91 | 0.58 | 2.07 | 0.43 |
| **Fear** | 2.48 | 0.42 | 1.95 | 0.36 | 1.97 | 0.32 | 1.46 | 0.33 |
| **Happiness** | 0.85 | 0.22 | 2.03 | 0.43 | 0.76 | 0.20 | 2.25 | 0.55 |
| **Sadness** | 6.36 | 0.73 | 4.13 | 0.52 | 7.66 | 0.94 | 5.65 | 0.94 |
| **Surprise** | 3.71 | 0.67 | 2.65 | 0.48 | 3.14 | 0.40 | 2.20 | 0.52 |
| **Neutral** | 23.45 | 1.25 | 28.12 | 1.20 | 23.78 | 0.94 | 27.86 | 1.14 |
| **Negative** | 3.74 | 0.32 | 3.34 | 0.36 | 3.73 | 0.36 | 3.09 | 0.43 |
| **Positive** | 2.28 | 0.34 | 2.34 | 0.31 | 1.95 | 0.26 | 2.23 | 0.40 |
|  |  |  |  |  |  |  |  |  |
| **Reaction time [ms]** |  |  |  |  |  |  |  |  |
| **Anger** | 1983.28 | 66.19 | 1966.74 | 52.99 | 2012.20 | 65.32 | 2018.54 | 67.88 |
| **Disgust** | 2014.56 | 62.05 | 1990.90 | 75.41 | 2074.56 | 63.44 | 1999.04 | 64.11 |
| **Fear** | 2335.82 | 77.36 | 2139.62 | 58.29 | 2494.83 | 69.50 | 2286.54 | 74.14 |
| **Happiness** | 1938.53 | 55.79 | 1802.38 | 50.71 | 1992.99 | 77.21 | 1868.41 | 68.75 |
| **Sadness** | 1663.86 | 38.83 | 1631.71 | 46.77 | 1717.33 | 59.90 | 1646.14 | 47.92 |
| **Surprise** | 1958.27 | 56.10 | 1747.58 | 44.57 | 1970.65 | 73.35 | 1877.41 | 78.73 |
| **Neutral** | 1878.87 | 70.23 | 1714.46 | 73.18 | 1874.88 | 52.50 | 1725.38 | 69.79 |
| **Negative** | 1999.38 | 45.69 | 1932.24 | 41.83 | 2074.73 | 51.68 | 1987.57 | 52.91 |
| **Positive** | 1948.40 | 48.67 | 1774.98 | 38.08 | 1981.82 | 60.30 | 1872.91 | 65.37 |
|  |  |  |  |  |  |  |  |  |
| **Response bias** |  |  |  |  |  |  |  |  |
| **Anger** | 0.75 | 0.04 | 0.71 | 0.03 | 0.81 | 0.03 | 0.78 | 0.03 |
| **Disgust** | 0.81 | 0.04 | 0.80 | 0.04 | 0.79 | 0.03 | 0.84 | 0.03 |
| **Fear** | 0.81 | 0.03 | 0.85 | 0.02 | 0.83 | 0.03 | 0.87 | 0.03 |
| **Happiness** | 0.93 | 0.01 | 0.84 | 0.03 | 0.94 | 0.01 | 0.84 | 0.03 |
| **Sadness** | 0.54 | 0.04 | 0.69 | 0.03 | 0.44 | 0.06 | 0.63 | 0.04 |
| **Surprise** | 0.75 | 0.04 | 0.82 | 0.03 | 0.76 | 0.03 | 0.85 | 0.03 |
| **Neutral** | -0.05 | 0.05 | -0.28 | 0.05 | -0.02 | 0.04 | -0.20 | 0.05 |
| **Negative** | 0.73 | 0.02 | 0.76 | 0.02 | 0.72 | 0.02 | 0.78 | 0.02 |
| **Positive** | 0.84 | 0.02 | 0.83 | 0.02 | 0.85 | 0.02 | 0.85 | 0.02 |
|  |  |  |  |  |  |  |  |  |
| **Target sensitivity** |  |  |  |  |  |  |  |  |
| **Anger** | 0.90 | 0.01 | 0.87 | 0.01 | 0.90 | 0.01 | 0.87 | 0.01 |
| **Disgust** | 0.90 | 0.01 | 0.85 | 0.01 | 0.90 | 0.01 | 0.88 | 0.01 |
| **Fear** | 0.87 | 0.01 | 0.87 | 0.01 | 0.86 | 0.01 | 0.87 | 0.01 |
| **Happiness** | 0.86 | 0.01 | 0.87 | 0.01 | 0.86 | <0.01 | 0.88 | 0.01 |
| **Sadness** | 0.90 | 0.01 | 0.89 | 0.01 | 0.91 | <0.01 | 0.89 | 0.01 |
| **Surprise** | 0.89 | 0.01 | 0.89 | 0.01 | 0.90 | 0.01 | 0.89 | 0.01 |
| **Neutral** | 0.84 | 0.01 | 0.85 | 0.01 | 0.80 | 0.02 | 0.82 | 0.02 |
| **Negative** | 0.89 | 0.01 | 0.87 | 0.01 | 0.89 | 0.01 | 0.88 | 0.01 |
| **Positive** | 0.88 | 0.01 | 0.88 | 0.01 | 0.88 | 0.00 | 0.88 | 0.01 |

Table S2. Correlations change-from-baseline scores between emotional cognitive measures and depression scores, controlling for baseline. Concurrent analysis.

|  |  | |  | **QIDS**  **6 weeks**  **minus baseline** |
| --- | --- | --- | --- | --- |
| FERT Accuracy Positive  Difference (6w – b)  6 weeks minus baseline | **Escitalopram** | Correlation | | -0.110 |
|  |  | Significance (2-tailed) | | 0.577 |
|  |  | df | | 26 |
|  | **Psilocybin** | Correlation | | -0.112 |
|  |  | Significance (2-tailed) | | 0.579 |
|  |  | df | | 25 |
| FERT Accuracy Negative  Difference (6w – b)  6 weeks minus baseline | **Escitalopram** | Correlation | | 0.184 |
|  |  | Significance (2-tailed) | | 0.349 |
|  |  | df | | 26 |
|  | **Psilocybin** | Correlation | | -0.123 |
|  |  | Significance (2-tailed) | | 0.542 |
|  |  | df | | 25 |
| FERT Misclassifications positive as negative  Difference (6w – b)  6 weeks minus baseline | **Escitalopram** | Correlation | | 0.250 |
|  |  | Significance (2-tailed) | | 0.200 |
|  |  | df | | 26 |
|  | **Psilocybin** | Correlation | | 0.182 |
|  |  | Significance (2-tailed) | | 0.364 |
|  |  | df | | 25 |
| FERT Misclassifications negative as positive  Difference (6w – b)  6 weeks minus baseline | **Escitalopram** | Correlation | | -0.115 |
|  |  | Significance (2-tailed) | | 0.559 |
|  |  | df | | 26 |
|  | **Psilocybin** | Correlation | | 0.301 |
|  |  | Significance (2-tailed) | | 0.127 |
|  |  | df | | 25 |

Table S3. Correlations change-from-baseline scores between emotional cognitive measures and depression scores at one-month follow-up, controlling for baseline. Longitudinal analysis.

|  |  | |  | **QIDS**  **1month FO**  **minus baseline** |
| --- | --- | --- | --- | --- |
| FERT Accuracy Positive  Difference (6w – b)  6 weeks minus baseline | **Escitalopram** | Correlation | | -0.387 |
|  |  | Significance (2-tailed) | | 0.092 |
|  |  | df | | 18 |
|  | **Psilocybin** | Correlation | | 0.095 |
|  |  | Significance (2-tailed) | | 0.700 |
|  |  | df | | 17 |
| FERT Accuracy Negative  Difference (6w – b)  6 weeks minus baseline | **Escitalopram** | Correlation | | 0.146 |
|  |  | Significance (2-tailed) | | 0.538 |
|  |  | df | | 18 |
|  | **Psilocybin** | Correlation | | -0.391 |
|  |  | Significance (2-tailed) | | 0.098 |
|  |  | df | | 17 |
| FERT Misclassifications positive as negative  Difference (6w – b)  6 weeks minus baseline | **Escitalopram** | Correlation | | 0.498 |
|  |  | Significance (2-tailed) | | 0.025 |
|  |  | df | | 18 |
|  | **Psilocybin** | Correlation | | -0.195 |
|  |  | Significance (2-tailed) | | 0.423 |
|  |  | df | | 17 |
| FERT Misclassifications negative as positive  Difference (6w – b)  6 weeks minus baseline | **Escitalopram** | Correlation | | -0.078 |
|  |  | Significance (2-tailed) | | 0.744 |
|  |  | df | | 18 |
|  | **Psilocybin** | Correlation | | 0.438 |
|  |  | Significance (2-tailed) | | 0.061 |
|  |  | df | | 17 |

Table S4. Sensitivity analysis. Correlations change-from-baseline scores between emotional cognitive measures and depression scores at one-month follow-up, controlling for baseline. Per protocol participants only. Concurrent analysis.

|  |  |  | **QIDS**  **6 weeks**  **minus baseline** |
| --- | --- | --- | --- |
| FERT Accuracy Positive  Difference (6w – b)  6 weeks minus baseline | **Escitalopram** | Correlation | -0.007 |
|  |  | Significance (2-tailed) | 0.976 |
|  |  | df | 18 |
|  | **Psilocybin** | Correlation | -0.240 |
|  |  | Significance (2-tailed) | 0.270 |
|  |  | df | 21 |
| FERT Accuracy Negative  Difference (6w – b)  6 weeks minus baseline | **Escitalopram** | Correlation | 0.221 |
|  |  | Significance (2-tailed) | 0.348 |
|  |  | df | 18 |
|  | **Psilocybin** | Correlation | -0.228 |
|  |  | Significance (2-tailed) | 0.183 |
|  |  | df | 21 |
| FERT Misclassifications positive as negative  Difference (6w – b)  6 weeks minus baseline | **Escitalopram** | Correlation | 0.143 |
|  |  | Significance (2-tailed) | 0.549 |
|  |  | df | 18 |
|  | **Psilocybin** | Correlation | 0.242 |
|  |  | Significance (2-tailed) | 0.267 |
|  |  | df | 21 |
| FERT Misclassifications negative as positive  Difference (6w – b)  6 weeks minus baseline | **Escitalopram** | Correlation | -0.313 |
|  |  | Significance (2-tailed) | 0.179 |
|  |  | df | 18 |
|  | **Psilocybin** | Correlation | 0.362 |
|  |  | Significance (2-tailed) | 0.089 |
|  |  | df | 21 |

Table S5. Sensitivity analysis. Correlations change-from-baseline scores between emotional cognitive measures and depression scores at one-month follow-up, controlling for baseline. Per protocol participants only. Longitudinal analysis.

|  |  |  | **QIDS**  **1month FO**  **minus baseline** |
| --- | --- | --- | --- |
| FERT Accuracy Positive  Difference (6w – b)  6 weeks minus baseline | **Escitalopram** | Correlation | -0.405 |
|  |  | Significance (2-tailed) | 0.151 |
|  |  | df | 12 |
|  | **Psilocybin** | Correlation | 0.439 |
|  |  | Significance (2-tailed) | 0.089 |
|  |  | df | 17 |
| FERT Accuracy Negative  Difference (6w – b)  6 weeks minus baseline | **Escitalopram** | Correlation | 0.232 |
|  |  | Significance (2-tailed) | 0.426 |
|  |  | df | 12 |
|  | **Psilocybin** | Correlation | -0.173 |
|  |  | Significance (2-tailed) | 0.521 |
|  |  | df | 14 |
| FERT Misclassifications positive as negative  Difference (6w – b)  6 weeks minus baseline | **Escitalopram** | Correlation | 0.490 |
|  |  | Significance (2-tailed) | 0.075 |
|  |  | df | 12 |
|  | **Psilocybin** | Correlation | -0.471 |
|  |  | Significance (2-tailed) | 0.065 |
|  |  | df | 14 |
| FERT Misclassifications negative as positive  Difference (6w – b)  6 weeks minus baseline | **Escitalopram** | Correlation | -0.384 |
|  |  | Significance (2-tailed) | 0.175 |
|  |  | df | 12 |
|  | **Psilocybin** | Correlation | 0.483 |
|  |  | Significance (2-tailed) | 0.058 |
|  |  | df | 14 |
